# Exploratory study on the effect of osteoactivin on muscle regeneration in a rat volumetric muscle loss model

**DOI:** 10.1371/journal.pone.0175853

**Published:** 2017-04-20

**Authors:** Jinjin Ma, Andrew R. Baker, Anthony Calabro, Kathleen A. Derwin

**Affiliations:** Department of Biomedical Engineering, Cleveland Clinic, Cleveland, Ohio, United States of America; University of Minnesota Medical Center, UNITED STATES

## Abstract

Wounds causing extensive injury loss of muscle, also known as volumetric muscle loss (VML), are frequently associated with high-energy civilian trauma and combat-related extremity injuries. Currently, no effective clinical therapy is available for promoting de novo muscle tissue regeneration to restore muscle function following VML. Recent studies have shown evidence that osteoactivin (OA), a transmembrane glycoprotein, has the ability to prevent skeletal muscle atrophy in response to denervation. Therefore the objective of this study is to investigate the potential regenerative effect of OA embedded and delivered via a cross-linked gelatin hydrogel within a volumetric tibialis anterior muscle defect in a rat model. After 4 weeks, however, no evidence for muscle formation was found in defects treated with either low (5 μg/ml) or high (50 μg/ml) OA. It is possible that a different delivery scaffold, delivery kinetics, or OA concentration may have yielded an alternate outcome, or it is also possible that the spaciostructural environment of VML, or the local (versus systemic) delivery of OA, simply does not support any potential regenerative activity of OA in VML. Together with prior work, this study demonstrates that an efficacious and scalable therapy for regenerating muscle volume and function in VML remains a veritable clinical challenge worthy of continued future research efforts.

## Introduction

Wounds causing extensive damage and loss of muscle, also known as volumetric muscle loss (VML), are frequently associated with high-energy civilian trauma and combat-related extremity injuries. These injuries commonly have a high projected healthcare cost and affect the quality of many patients’ lives. It is estimated that patients with VML in extremity injuries have an averaged healthcare cost of $80,000–90,000 the first two years, and a projected lifetime healthcare cost of $160,000–510,000 [1]. Currently surgical reconstruction (i.e., free muscle transfer) is performed with the intent to restore partial function. However, donor site morbidity and chronic pain are frequently associated with the reconstruction. Moreover, many patients eventually face or choose amputation due to the functional deficits from the injury site [2]. No effective clinical therapy is available for promoting de novo muscle tissue regeneration following VML. Clearly, there exists a need for improved strategies to foster new muscle formation to restore the muscle function in patients with VML.

Currently, ongoing efforts are being made to develop tissue engineering and regenerative medicine strategies for regenerating appreciable and functional muscle tissue in the setting of VML. The majority of work has explored treatment of VML with decellularized or devitalized extracellular matrix (ECM) scaffold implants, derived from small intestine submucosa (SIS) [3–7], urinary bladder wall (UBM) [6, 8, 9], or muscle ECM [8, 10–12]. Currently, ECM scaffolds derived from SIS (Restore, DePuy Orthopaedics, Inc, Warsaw, IN) and UBM (Matristem Devices, ACell, Columbia, MD) have been FDA cleared, and there is an ongoing clinical study to investigate the efficacy of restoring muscle function using SIS and UBM ECM in VML patients [5, 13, 14]. Although ECMs possess many characteristics that could potentially promote muscle regeneration, investigations of ECM scaffolds in animal models of VML have shown a spectrum of results from abundant muscle regeneration [3, 7] to absent muscle deposition in the defect [4, 15, 16]. Overall most studies have shown that at prolonged time points, ECMs do not promote appreciable muscle fiber regeneration in vivo, particularly in regions of the defect remote from the remaining muscle bed [5, 6, 8, 12, 14].

More recently, vital minced muscle autograft has been shown in animal models to promote muscle regeneration in a VML defect [17, 18]. Although these findings are quite promising, the widespread application of vital minced muscle autograft therapy is inherently limited due to graft availability and donor site morbidity. Other ongoing efforts are investigating mesenchymal stem cell (MSC) based therapies for VML, where MSCs may participate directly in tissue regeneration as well as play a “trophic” or supportive role in the process. Results of cell-therapy approaches to date have shown varying levels of functional muscle recovery after repair, however, little to no neo-muscle was found in the defects treated with MSCs [19–21]. Together, these studies demonstrate that an efficacious and scalable therapy for regenerating muscle volume and function in VML is still lacking.

To address this unmet need, we propose to investigate a novel small molecule therapeutic approach to muscle regeneration. Osteoactivin (OA), also known as glycoprotein non-melanoma clone B (GPNMB), is a transmembrane glycoprotein expressed in numerous cells and tissues. Recent studies have shown it has the ability to regulate cell proliferation, adhesion, differentiation and synthesis of ECM proteins [22–26]. Overexpression of OA in transgenic mice has been shown to prevent skeletal muscle atrophy in response to denervation, as well as to provide a neuroprotective function in amyotrophic lateral sclerosis models [27]. In light of these findings, we speculate that directly delivering OA to a VML defect may guide the activity of recruited host cells such as MSCs, muscle progenitor cells and inflammatory cells and further orchestrate the spatiotemporal events needed for muscle regeneration. As such, the objective of this study is to investigate the potential regenerative effect of osteoactivin (OA) embedded and delivered via a cross-linked gelatin hydrogel within a volumetric tibialis anterior (TA) muscle defect in a rat model. We hypothesize that defects treated with OA gels will demonstrate greater neo-muscle formation than the contralateral repairs with gels alone. If successful, this new strategy would be a translatable and less morbid approach than current clinical standard of care or other investigational approaches for volumetric muscle regeneration using vital autografts or autologous MSCs.

## Materials and methods

### Study design

Human recombinant OA was purchased from R&D systems (Minneapolis, MN). Lyophilized tyramine substituted gelatin hydrogel (gel) was produced in our laboratory according to a previously published protocol [28]. Cuboidal shaped gel constructs with no OA (gel only, control), low dose OA (5 μg/ml) and high dose OA (50 μg/ml) were fabricated to investigate the in vitro release characteristics and in vivo regenerative effect of OA in a rat TA muscle VML model. Fifteen male Sprague Dawley rats (200–250 g) were used for the in vivo study (Harlan Laboratories, Indianapolis, IN). Twelve rats were randomly divided into low dose OA (n = 6) and high dose OA (n = 6) groups. A near full-thickness volumetric defect (12x4x4 mm) was created in the middle third of the left TA muscle in each animal and treated with a size-matched gel construct containing either low or high OA. As a control, the same defect was created in the right contralateral TA muscle and treated with a gel only construct in each animal. The rats were euthanized at 2 and 4 weeks (N = 3/group/time point). The TA muscle from both legs was explanted and evaluated histologically for neo-muscle formation and pan-macrophage density as the primary outcomes. Muscle size and the resorption of the gel constructs were also assessed. An additional rat (n = 1) was used to generate a surgical repair for evaluation at time zero (the rat was euthanized immediately after surgery was completed). Two rats (N = 1 at 2 weeks and N = 1 at 4 weeks) served as gender and age matched controls for muscle growth (rats were euthanized without performing surgery). Detailed methods are described below.

### Gel constructs preparation

**Fig 1:**
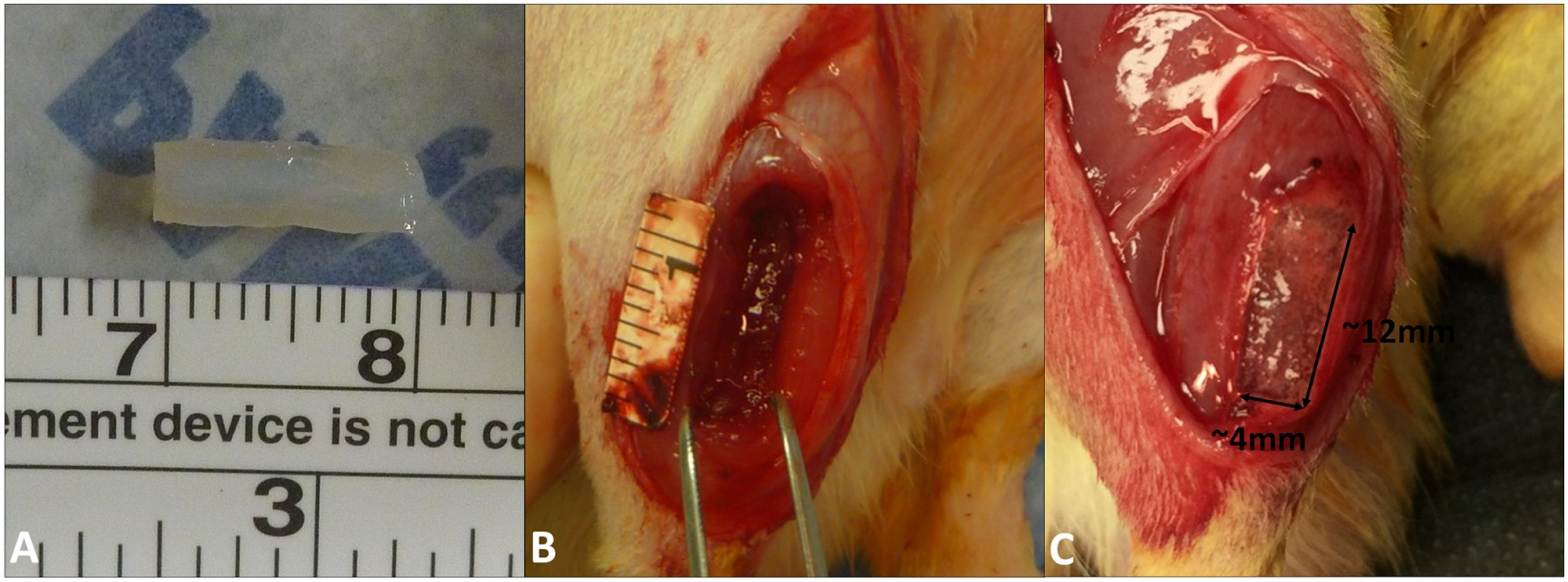
The fabrication of gel constructs and VML injury and treatment in the rat TA muscle. (**A**) Cross-linked gel constructs before in vitro testing or in vivo implantation. (**B**) A 12x4x4mm near full-thickness VML defect was created in the middle-third of the rat TA muscle. (**C**) A size-matched gel construct (low OA, high OA or gel only) was placed in the defect for treatment.

OA was dissolved in ultrapure water to create a stock solution (100 μg/ml). Lyophilized gel was hydrated at 50 mg/ml in 0.5X PBS overnight at 37°C. Gel solutions were partially evaporated and stock OA and horseradish peroxidase (HRP, 1 U/μl) added. Ultrapure water was added to bring the gel solution back to its original volume with the final solution concentrations 50 mg/ml gel, 10 U/ml HRP and either 5 μg/ml (low) or 50 μg/ml (high) OA. The resulting solutions were pipetted into customized molds with dimensions of 12x4x4 mm (192 μl). The filled mold was frozen at -20°C overnight. For cross-linking, frozen gel constructs were aseptically pressed out of the mold and submerged into 0.3% H_2_O_2_ in 0.2 X PBS solutions for approximately 5 minutes. Constructs were crosslinked immediately before in vitro testing or in vivo implantation (Fig 1A).

### In vitro OA release

The release of OA from gel constructs was studied by in vitro by incubation in RPMI 1640 Medium with L-glutamine (Cell Services-Media Core, Cleveland Clinic). Gel only, low OA, and high OA gels (N = 3/group) were placed in 24-well plates with 2 ml (~10 times that of the gel volume) of medium in each well. The plates were incubated at 37°C and 5%CO_2_ for 5 days. The amount of OA in the supernatant was quantified using the human OA/Gpnmb DuoSet^®^ ELISA development system (R&D Systems, Minneapolis, MN) according to the manufacturer’s recommendation. At 5 days, intact gels were collected, snap-frozen in cold isopentane, and stored at -20°C. Additional gels from gel only, low OA, and high OA gel groups were made and snap-frozen for time-zero histology control. Cryosections (8 μm, multiple adjacent sections) were obtained to visualize OA in the gel at time zero and after 5 days of incubation via immunohistochemistry. The primary antibody used was goat anti-human OA (R&D Systems) with a concentration of 3 μg/ml in PBS. ImmPRESS^™^ HRP anti-goat IgG polymer detection kit (Vector Labs, Burlingame, CA) and ImmPACT DAB Peroxidase substrate (Vector Labs) were used for detection.

### In vivo VML injury and treatment

#### Surgical procedure

All procedures were performed in accordance with the National Institutes of Health (NIH) guidelines for care and use of laboratory animals and were performed as approved by the Institutional Animal Care and Use Committee (IACUC) at the Cleveland Clinic. Each rat was anesthetized with an intraperitoneal injection of ketamine, xylazine, and acepromazine (30/6/1 mg/kg). The surgical procedure for creating VML in the rat TA muscle was performed as described in Wu et al., 2012 [29]. Briefly, a 20 mm longitudinal incision was created along the lateral aspect of the lower leg. With the TA muscle exposed, a longitudinal cut towards posterior of the muscle was made on the overlying fascia. Cautery was used to demarcate the four corners of a 12x4 mm defect area in the middle third of the TA muscle. Two 12 mm long and 4 mm deep parallel incisions, separated by 4 mm distance, were created using a custom-made double blade scalpel. A 4 mm deep horizontal incision was then created to connect the proximal end of the two parallel incisions. A customized U-shaped wire was inserted into the proximal cut and pulled distally to complete the 4 mm deep cut, and spring scissors were used to connect the distal ends of the two parallel incisions. The result was a 12x4x4 mm near full-thickness defect in the middle-third of the TA muscle (Fig 1B). In all groups, a size-matched gel only construct was placed in the defect in the right TA muscle and a size-matched OA-gel construct (low OA or high OA) was placed in the defect in the left TA muscle (Fig 1C). The fascia was re-approximated over the gel-filled defects using continuous 6–0 Prolene suture (Ethicon, Blue Ash, OH). Skin was closed using running subcuticular 5–0 Vicryl suture (Ethicon). While the animals were still under anesthesia, an E-collar was fitted to the rats to limit their access to the incision during the first week of the post-operative period. All animals were given buprenorphine hydrochloride (0.02–0.05 mg/kg) twice a day for 3 days and acetaminophen in their water (2 mg/ml) for 7 days for post-operative pain management. Animals were euthanized at 2 weeks (n = 6) and 4 weeks (n = 6) by carbon dioxide asphyxiation.

#### Histology analysis

*Histology and immunohistochemistry*: The entire TA muscle from each leg was harvested en bloc, snap-frozen in cold isopentane and stored at -20°C. Serial coronal cryosections (8 μm) were cut at 0.5 mm intervals step-wise from the anterior edge of the TA muscle. Sections from 3–7 step levels fell within the muscle defect region of each TA muscle. At each step level, one section was stained with hematoxylin and eosin (H&E) for general morphologic observations; adjacent sections were immunostained for PAX7 (satellite cells), F1.652 (embryonic myosin) and CD68 (Pan macrophages). Specifically, all frozen sections were fixed in neutral buffered formalin for 5 min. For immunostaining, sections were rinsed three times 5 min with PBS and then incubated with 0.3% H_2_O_2_ (ThermoFisher Scientific, Waltham, MA) in distilled water for 10 minutes at room temperature to inhibit endogenous peroxidase activity. Sections were rinsed again in PBS, and then incubated in 0.2% Triton X-100 in PBS for 5 min. Nonspecific binding of the primary antibody was blocked using 2.5% normal horse serum in PBS for 30 minutes. After blocking, sections were incubated either in F1.652 (Developmental Studies Hybridoma Bank, Iowa City, IA) at 1:10 dilutions, mouse anti rat PAX7 (Developmental Studies Hybridoma Bank) at 1:10 dilutions or mouse anti rat CD68 (Bio-Rad, Raleigh, NC) at 1:100 dilutions in a humidified chamber at room temperature for 1h. F1.652 and CD68 staining was visualized with ImmPRESS^™^ HRP rat adsorbed anti-mouse IgG polymer detection kit (Vector Labs) and ImmPACT DAB Peroxidase substrate (Vector Labs). After rinsing in PBS for 5 min, F1.652 and CD68 sections were submerged in hematoxylin for 1 minute (for visualization of cell nuclei) and then mounted. F1.652 and CD68 sections were scanned with a Leica SCN400F (Leica Microsystems, GmbH, Wetzlar, Germany) for observation and quantitative analysis. PAX7 staining was visualized with fluorophore-conjugated secondary antibody (546 goat anti-mouse IgG, Invitrogen, 1:500). 4',6-diamidino-2-phenylindole (DAPI) and wheat germ agglutinin (WGA) were used for staining nuclei and extracellular matrix. PAX7 sections were imaged on a Leica DMR inverted microscope equipped with Qimaging Retiga EXi camera.

*Macrophage scoring*: CD68 stained sections from all samples from each group were used to semi-quantitatively score the density of CD68+ cells in the defect in a blinded fashion. In each sample, the step-level section that had the largest *defect* area among all available sections was used for scoring. Semi-quantitative evaluation CD68+ cell density at the periphery and the center of the gel was performed by one study investigator. A 7 point scale was used to grade CD68+ cell density, where, Grade 1: minimal/none, Grade 2: Scant; Grade 3: Modest; Grade 4: Modest to moderate; Grade 5: moderate; Grade 6: moderate to heavy; Grade 7: heavy.

*Cross-sectional area (CSA) of the TA muscle and remaining gel in the defect*: The CSA of TA muscle was estimated from *one* section from each sample from each group, specifically at the step-level section located 5.5 mm deep from the anterior edge of the muscle, because this was the location of the largest *muscle* cross-sectional area. Both of the TA muscles from one age and gender matched rat at 2 and 4 weeks were used to estimate the normal cross-sectional area of the rat TA muscle at the same step level (n = 2/time point). *Muscle* CSA measures were repeated three times on any given 5.5 mm step level section, and these repeats were averaged as an estimate of CSA for that muscle. The CSA *of the remaining gel* was estimated from *three* sections from each sample in each group, specifically at the step-level section where the largest gel cross-section was observed, as well as the two adjacent step-levels. Measurements of gel area were made in triplicate on each section, and the average value from the three sections was used as an estimate of gel CSA for that sample. All muscle and gel perimeter tracing and area calculations were performed with Aperio Image Scope software (Version 12.0.1.5027, Leica Biosystems Inc., Buffalo Grove, IL).

### Statistical analysis

Wilcoxon signed-rank test was performed to examine the effect of OA treatment compared to gel only treatment on the histology score of CD68+ cells and on gel CSA at 2 weeks and 4 weeks. A *p* value of ≤ 0.05 was considered significant. Statistical analysis was performed using SigmaPlot 13.0.

## Results

### *In vitro* OA release

**Fig 2:**
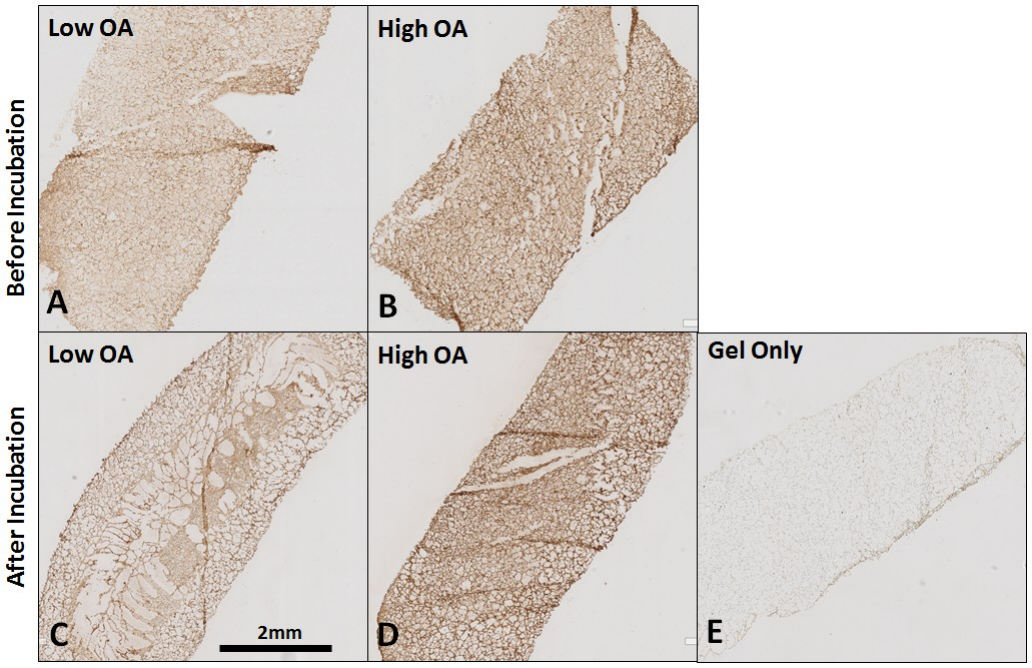
Representative sections of gels before (**A**, **B**) and after 5 days (**B**-**E**) in vitro incubation showed OA was detected in gels in both low OA (**A**, **C**) and high OA (**B**, **D**) groups while no OA in gel only group (**E**) Before and after incubation, the amount of OA in the high OA group (**B**, **D**) appeared higher than that in the low OA group (**A**, **C**). The amount of OA in each low or high OA group after incubation appeared to be similar in the gels before incubation. Scale bar = 2 mm applies to all images.

Prior to incubation, OA was uniformly distributed in both the low and high OA gels (Fig 2A and 2B). The amount of OA in high OA group (Fig 2B) appeared higher than that in the low OA group (Fig 2A). After 5 days of in vitro incubation, the amount of OA in both the low (Fig 2C) and high (Fig 2D) OA groups appeared similar to the amount of OA in the respective gels prior to incubation. Quantitatively, the total amount of OA released from the gel after 5 days of *in vitro* incubation was 0.036±0.003μg (3.6% of the total OA embedded in the gel) in low OA group and 0.75±0.282 μg (7.5% of the total OA embedded in the gel) in high OA group (S1 File). Since the amount of OA released after 5 days of *in vitro* incubation was less than 10% of the total OA embedded in the gel, the *in vitro* release kinetics of OA were not further studied. These data suggest that the release of substantive amounts of OA from the gel *in vivo* will require gel degradation.

### *In vivo* VML injury and treatment

#### General observation

**Fig 3:**
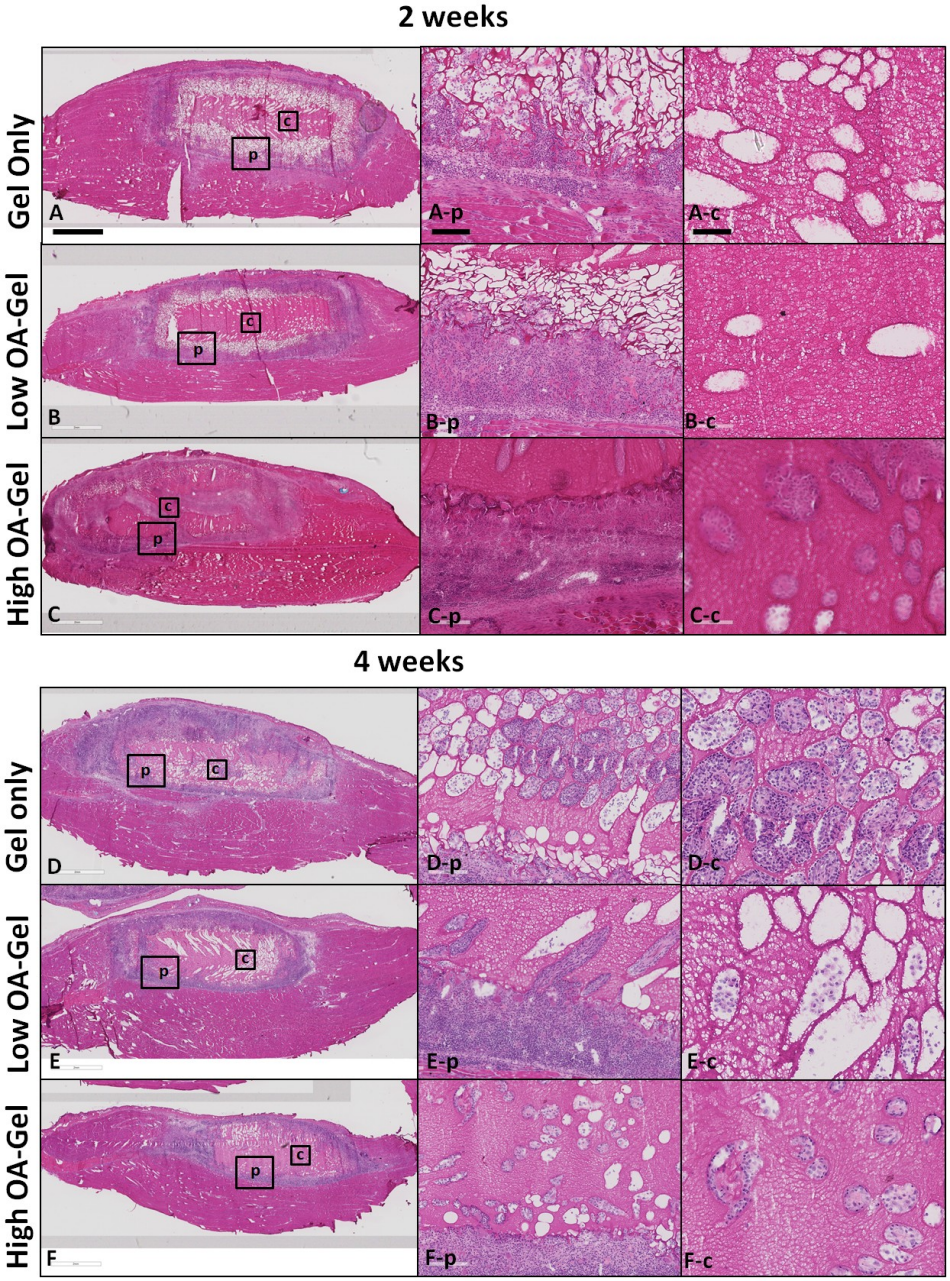
Representative sections of 2 week and 4 week TA muscle explants treated with gel only, low OA and high OA gels (H&E staining). In all groups at both time points, remnants of gel encapsulated by a thick cell layer (**A**-**E**), in which the majority of the cell population was inflammatory cells (**A-p** to **F-p**) was observed. No to scant cells were observed in the center of the gels at 2 weeks (**A-c** to **C-c**). At 4 weeks, a greater degree of cellular invasion into the center of the gels was observed (**D-c** to **F-c**) than at 2 weeks. Scale bar is 2mm in A-F, 200 μm in **A-p** through **F-p**, and 100 μm in **A-c** through **F-c**.

**Fig 4:**
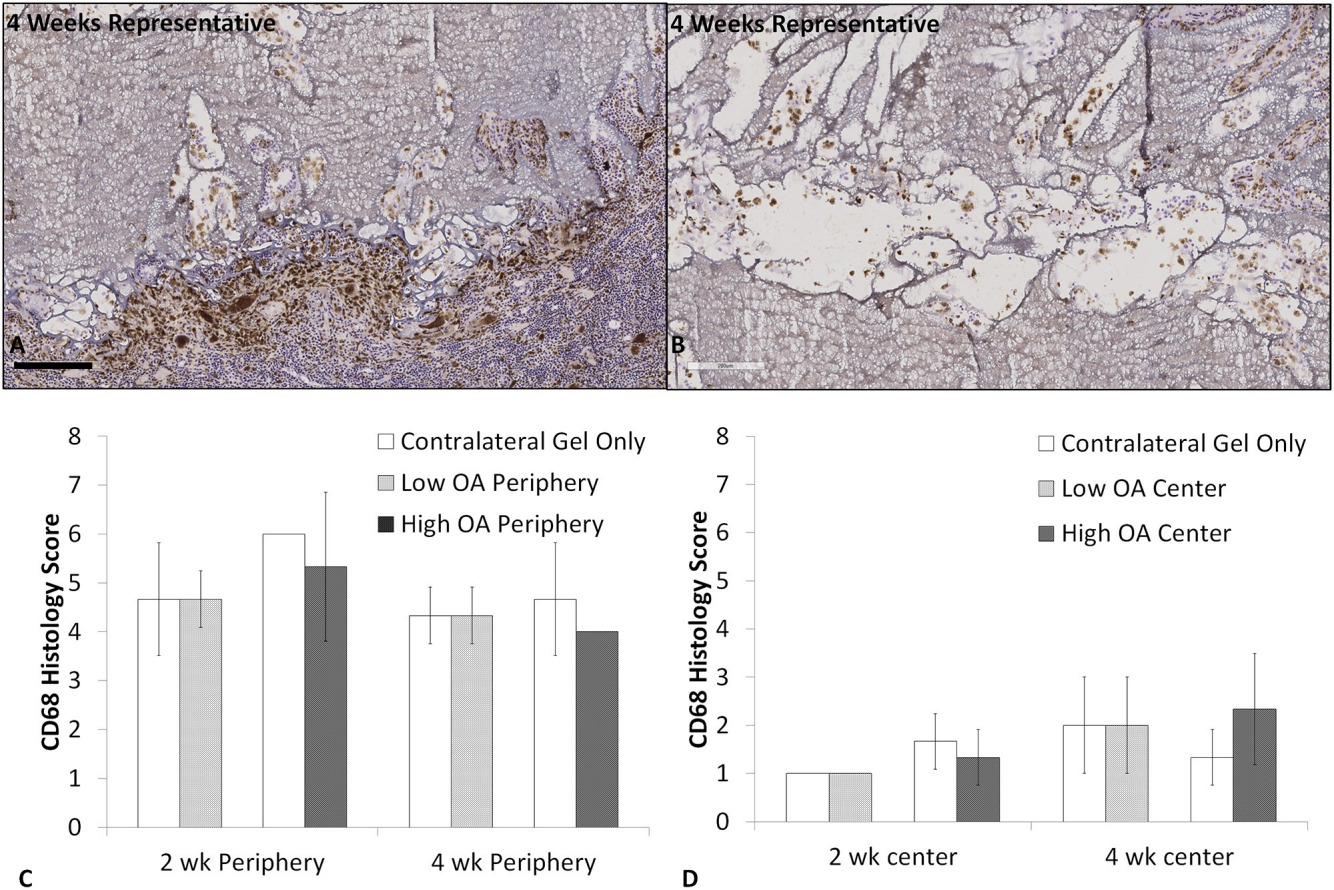
Representative sections from a 4 week gel-only repair showed that the majority of the cells encapsulating the gel (**A**) or within the gel (**B**) are CD68+ macrophages. CD68+ scoring showed no significant difference between OA treated (low or high OA) and contralateral (gel only) defects at 2 or 4 weeks at either the periphery (**C**) or center (**D**) of the gel. Scale bar = 200 μm in **A** and **B**.

In low OA, high OA and gel only control groups at 2 weeks (Fig 3A–3C) and 4 weeks (Fig 3D–3F), remnants of gel encapsulated by a thick cell layer in which the majority of the cell population was inflammatory cells was observed (Fig 3A-p to 3F-p). None to scant numbers of cells were observed in the center of the gels at 2 weeks (Fig 3A-c to 3C-c). At 4 weeks, a greater degree of cellular invasion into the center of the gels was observed (Fig 3D-c to 3F-c) than at 2 weeks (Fig 3A-c to 3C-c).

#### Macrophages

Immunohistochemistry analysis showed a high density of CD68+ macrophages encapsulating the gel (Fig 4A) and inside of the gel (Fig 4B). Scored CD68+ cell density (S1 File) was not significantly different between OA treated (low or high OA) and contralateral (gel only) defects at either 2 or 4 weeks, regardless of the location being evaluated (periphery or center of the gel) (Fig 4C and 4D).

#### PAX7+ satellite cells and muscle fibers

**Fig 5:**
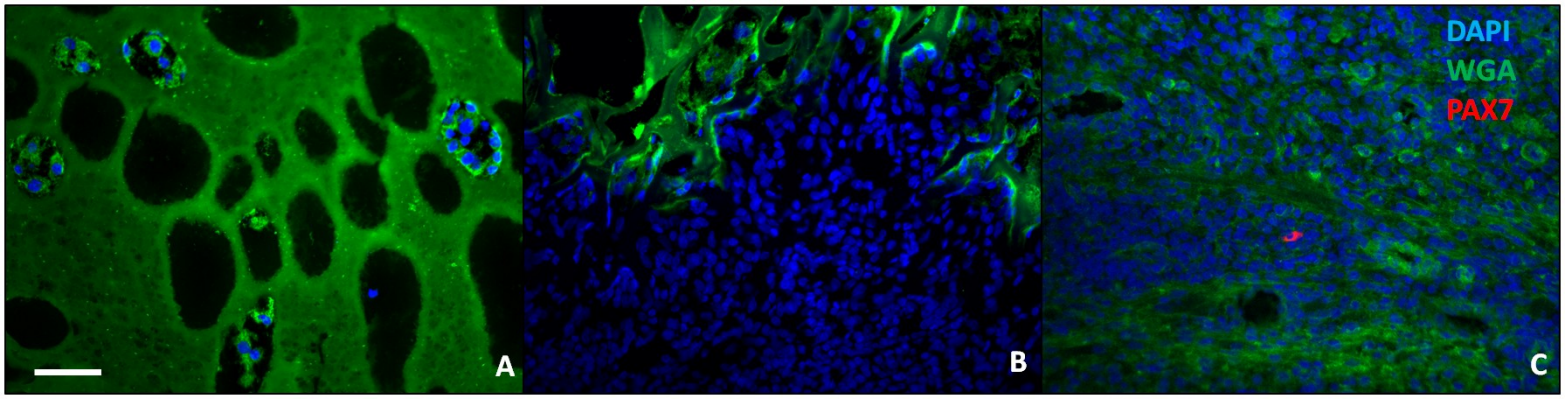
Representative sections from a 2 week TA muscle explants treated with low OA gel showed that no PAX7+ satellite cells were obsereved in the center of the defect (A) or at the edge of the defect (B). PAX7+ cells were occasionally observed in the host muscle in proximity to the defect edge, which is likely tissue that was injured during surgery (C). Scale bar = 100 μm.

**Fig 6:**
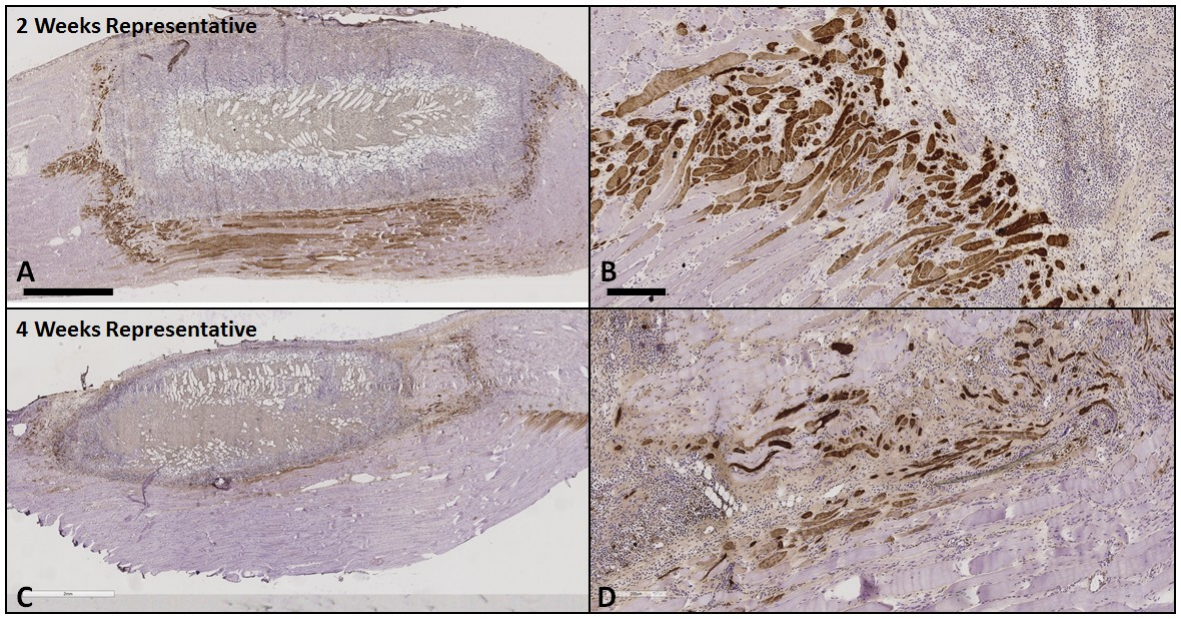
Represenative sections from a 2 week (**A** and **B**, gel only) and 4 week (**C** and **D**, gel only) TA muscle explant showed that embryonic myosin (f1.652+) was only observed in proximity to the edges but not within the defect, providing evidence for regenerating host muscle fibers that were injured during surgery but not neo-muscle formation in the defect. The density of f1.652+ fibers was observed to be higher at 2 weeks than 4 weeks. Scale bar = 2 mm in A and C. Scale bar = 200 μm in **B** and **D**.

PAX7+ satellite cells were not observed in either the center (Fig 5A) or at the edge of the defect (Fig 5B) in any sample regardless of treatment group or time point, so no statistical comparison between treatment groups could be performed. Occasionally PAX7+ cells were observed in the host muscle in proximity to the defect edge, which is likely tissue that was injured during surgery (Fig 5C). Similarly, muscle fibers staining for embryonic myosin (f1.652+) were not observed within the defect in any sample (Fig 6), so no statistical comparison between treatment groups could be performed. f1.652+ muscle fibers were observed in the host muscle in proximity to the defect edge (Fig 6), which are likely regenerating host muscle fibers that were injured during surgery. The density of f1.652+ muscle fibers in the host muscle surrounding the defect was observed to be higher at 2 weeks (Fig 6A and 6B) than 4 weeks (Fig 6C and 6D).

#### Muscle hypertrophy

**Fig 7:**
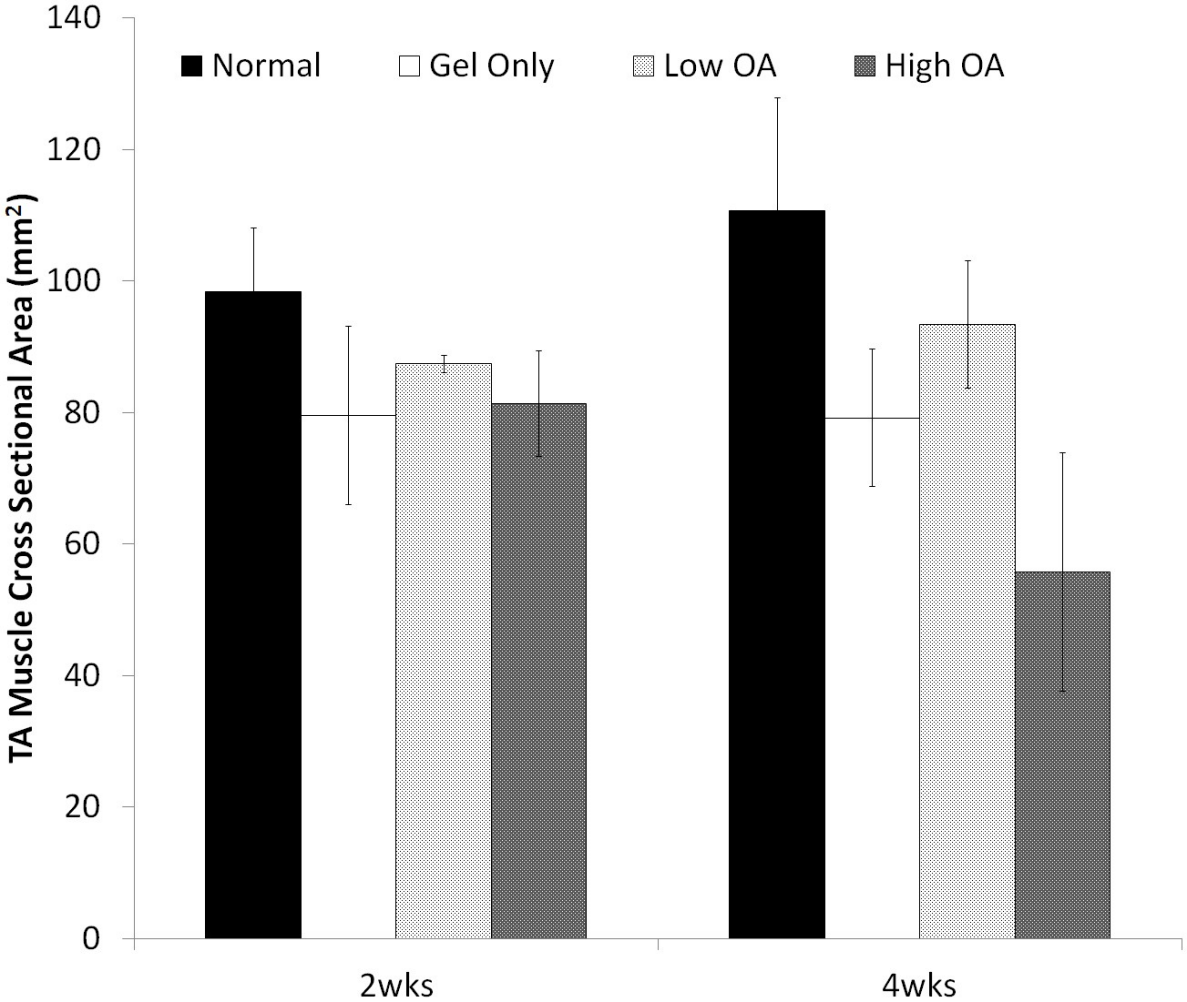
Measurements of TA muscle CSA at the step-level section located 5.5 mm deep from the anterior edge of the muscle at 2 weeks and 4 weeks. At 2 weeks the CSA of the TA muscles in the no treatment (gel only) and OA treatment groups was decreased to 63–93% of the CSA of the TA muscle from an age and gender matched normal rat at the same step-level. At 4 weeks the CSA of the TA muscles in the no treatment (gel only) and OA treatment groups was similarly decreased to 60–90% of the CSA of the TA muscle from an age and gender matched normal rat at the same step-level.

No statistical analysis was performed on muscle CSA due to the small sample size (N = 2) in each group (S1 File). Descriptively, at 2 weeks the CSA of the TA muscles in the gel only and OA treatment groups was decreased to 63–93% of the CSA of the TA muscle from an age and gender matched normal rat (Fig 7). At 4 weeks the CSA of the TA muscles in the gel only and OA treatment groups was similarly decreased to 60–90% of the CSA of the TA muscle from an age and gender matched normal rat (Fig 7).

#### Gel resorption

**Fig 8:**
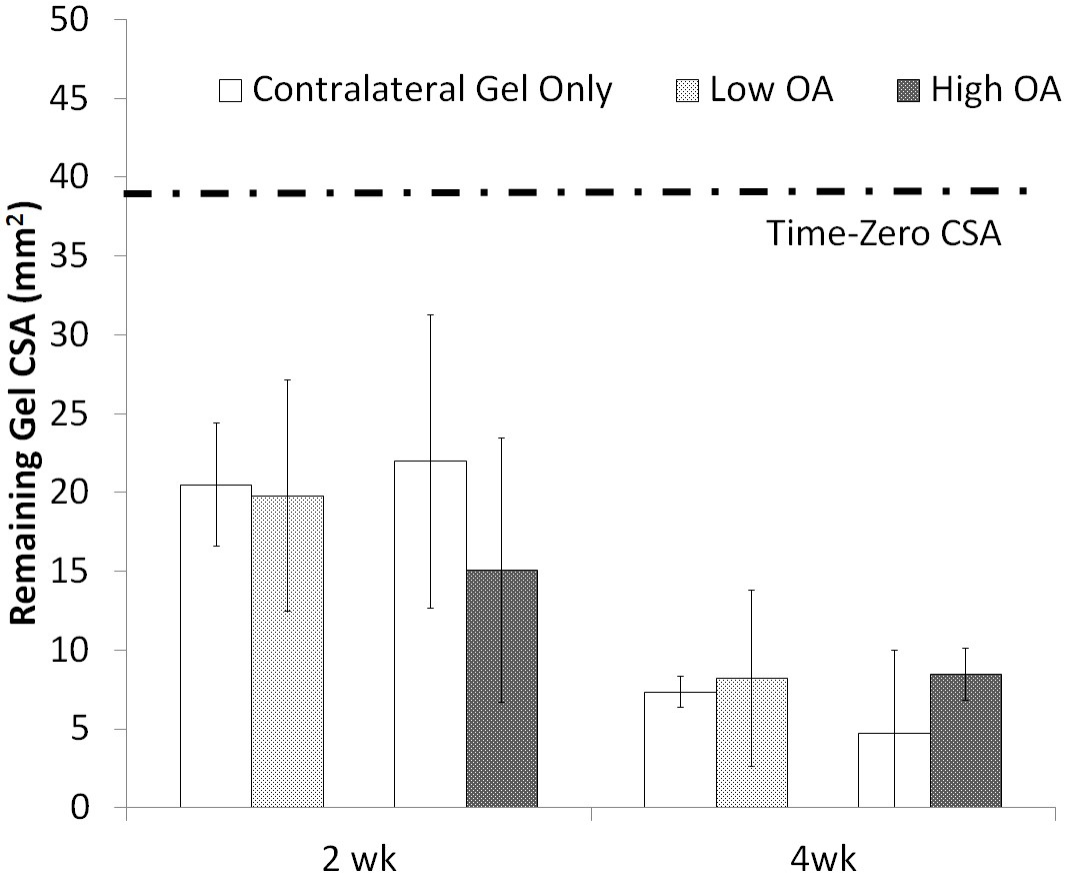
Analysis of gel resorption at 2 and 4 weeks. At implantation, the CSA of a prototypical gel was 39 mm^2^. Two weeks after the repair, the averaged CSA of the remaining gel was approximately half of its original cross-section (24%-80%) across all groups, and by four weeks the remaining gel was less than one quarter (4%-31%) of its original cross-section. No significant difference was found between the CSA of the remaining gel in muscles with treatment (low or high OA) and contralateral muscles with no treatment (gel only) at either 2 or 4 weeks.

At implantation, the CSA of a prototypical gel cast in 0.2X PBS was 39 mm^2^, which we acknowledge could have reduced in size after implantation in physiologic environment by as much as 30%, based on laboratory experiments equilibrating gels in 1X PBS for an hour. Two weeks after the repair, the average CSA of the remaining gel was approximately half of its pre-implantation cross-section (24%-80%) across all groups, and by four weeks the remaining gel was less than one quarter (4%-31%) of its pre-implantation cross-section (Fig 8). These data suggest degradation of the gel beyond any initial shrinkage that may have occurred. No significant difference was found between the CSA of the remaining gel in muscles with treatment (low or high OA) and contralateral muscles with gel only treatment at either 2 or 4 weeks (S1 File).

## Discussion

The goal of this study was to investigate the extent to which OA, embedded and delivered via a cross-linked gelatin hydrogel within a volumetric TA muscle defect in a rat model, could foster muscle regeneration. The defect created in the TA muscle was equivalent to more than 20% of the entire TA muscle mass, and this model is commonly chosen for VML studies. As expected, the TA muscles that had gel only treatment underwent no muscle formation within the defect. However, contrary to our hypothesis, TA muscle defects treated with either low or high OA also showed no muscle formation.

The powerful anabolic activity of OA in bone tissue formation and its regenerative effect in critically-sized bone defects has been extensively demonstrated [30]. Although OA hasn’t been broadly studied in muscle regeneration, studies have suggested at least a protective if not regenerative potential for OA in the setting of muscle injury. Ogawa et al. 2005 showed that the OA expression was increased in mouse skeletal muscle after denervation, and provided evidence to suggest that OA might function as an activator for infiltrating fibroblasts to produce collagen type I, MMP-3 and MMP-9 [27]. The authors suggest that an OA-mediated increase in MMPs in skeletal muscle might be useful for regeneration in denervated skeletal muscle, leading to compensation for the loss of muscle volume or protection of muscle fibers against injury after denervation. Furochi et al 2007 went on to show that overexpression of OA (in an OA-transgenic mouse model) protected skeletal muscle from severe degeneration of myofibers and fibrosis caused by long-term denervation (70–90 days) [31]. Further, in a distraction osteogenesis muscle injury model, overexpression of OA was associated with increased expression and levels of MMP-3 and MMP-9 and less collagen accumulation, suggesting OA may attenuate skeletal muscle fibrosis by promoting extracellular matrix degradation/remodeling [32].

The current investigation focused on muscle injury by means of VML, an injury also associated with inflammation and fibrosis, which are factors known to inhibit muscle regeneration [33, 34]. We speculated that the delivery of OA directly into the muscle defect might mitigate inflammation and fibrosis and facilitate neo-muscle formation. However, unlike prior studies where OA released from (glutaraldehyde) cross-linked hydrogels showed a dose dependent treatment effect on in vitro osteoblastic differentiation of mesenchymal cells [35], OA delivered to a muscle defect by means of a (hydrogen peroxide) cross-linked hydrogel in this study was not associated with an in vivo treatment effect. Our findings were unexpected in light of prior work showing a protective and possibly regenerative role for OA in muscle injury [7, 26, 27], but may possibly be explained by the following factors. First, muscle preservation in the context of OA over-expression was observed in denervation and distraction injury models, in which the entire muscle ECM was well preserved; whereas in the VML model, a portion of the muscle ECM and associated cells are removed and an artificial scaffold is required for OA delivery. Second, the denervation and distraction injury models were in mice systemically over-expressing OA, whereas in the VML model the OA was available only by means of local delivery to the injury site. Third, PAX7+ satellite cells which are known to be necessary for normal muscle regeneration [36] were not observed within the defects in any group at 2 or 4 weeks. It is possible that fibrous encapsulation around the gel (Fig 3) may have prevented the PAX7+ satellite cells migrating into the defect and therefore impeded any muscle regeneration, a mechanism recently discussed by [37]. Alternatively, it is possible that the absence of the PAX7+ satellite cells in the defect could be the consequence of macrophage mediated host response that favored fibrosis (migration of fibroblasts) over regeneration (migration of satellite cells). Hence, is possible that either the spaciostructural environment of VML, the slow release kinetics of OA from the degrading hydrogel or the localized delivery mechanism in the VML model simply did not support any potential regenerative activity of OA in this study.

Skeletal muscle is known to have a remarkable capacity to regenerate in cases of physical trauma involving minimal loss of tissue [38, 39]. However, with VML injury where a critical-sized muscle is lost, the remaining muscle mass is unable to regenerate appreciable amount of muscle and therefore fails to restore function [38, 39]. Despite considerable ongoing investigation, no definitive therapy for VML that regenerates or restores the loss of muscle mass has been developed. Most studies utilizing decellularized or devitalized ECMs showed no appreciable muscle regeneration in vivo [5, 6, 8, 12, 14]. Vital minced muscle autograft has shown some promise in promoting muscle regeneration in a VML defect [17, 18], yet widespread application of this therapy is inherently limited due to graft availability and donor site morbidity. Results of cell-therapy approaches to date have shown varying levels of functional muscle recovery after repair, however, little to no neo-muscle was found in the defects treated with MSCs [19–21]. Finally, our study investigated a novel small molecule therapeutic approach to muscle regeneration in VML, yet no muscle regeneration was demonstrated.

Several limitations are associated with this study. First, only two concentrations of OA were investigated, though these were logically selected based on OA treatment levels that had resulted in significant bone formation in a critically sized calvarial defect [30]. Second, the study endpoints were relatively short. The two-week time point was chosen so as to observe patterns of early macrophage infiltration, known to play an essential role in skeletal muscle regeneration after injury [40]. The four-week point was chosen because the regenerated muscle fibers could be expected by four weeks post-injury [36]. Since the density and pattern of the macrophages response (CD68+ cells) was not different between OA-treated and gel-only treated muscle defects, nor did OA-treatment mitigate hypertrophy in the injured muscles, nor did OA-treatment associate with any evidence of muscle formation in the defect, investigation at longer endpoints was nonsensical and not pursued. Further, the study has a low sample size. Our intent was to expand the sample size had evidence to support a role for OA treatment in muscle regeneration in the established VML model been demonstrated, but absent such evidence, further expansion of the study groups was also not pursued. Finally, we did not formally confirm that the OA was functionally active following hydrogen peroxide cross-linking into our hydrogel; however it is reasonable to assume that if OA released from glutaraldehyde cross-linked hydrogels remained biologically active [35], the much less harsh hydrogen peroxide cross-linking would not inactivate the molecule either.

To our knowledge, this is the first study to evaluate OA in VML. This preliminary investigation showed no evidence for muscle formation in a rat near-full thickness TA muscle defect model treated with OA delivered in a hydrogel construct. While it is possible that the spaciostructural environment of VML and/or the local (versus systemic) delivery of OA does not support any potential regenerative activity of OA in VML, it cannot be concluded from this study that OA therapy has no value for volumetric muscle regeneration. For example, it is possible that a different delivery scaffold, delivery kinetics, or OA concentration may have yielded an alternate outcome. It is also possible that co-delivery of OA with minced muscle autograft could stimulate more regeneration than minced muscle alone and thereby reduce the morbidity associated with autograft procurement by reducing the amount of autograft needed. Future work should investigate alternate approaches to OA therapy in an effort to address the veritable clinical challenge of identifying an efficacious and scalable therapy for regenerating muscle volume and function in VML.

## Supporting information

S1 File Raw data collection of OA in vitro release ELISA, TA muscle CSA, CD68 score and gel resorption. (XLSX)
